# Guiding Treatment Choices for Elderly Patients with Glioblastoma by a Comprehensive Geriatric Assessment

**DOI:** 10.1007/s11912-020-00951-6

**Published:** 2020-07-10

**Authors:** Carola Lütgendorf-Caucig, Christian Freyschlag, Eva Katharina Masel, Christine Marosi

**Affiliations:** 1 EBG MedAustron GmbH, Marie-Curie-Strasse 5, 2700 Wiener Neustadt, Austria; 2 Department of Neurosurgery, Medical University of Innsbruck, Innsbruck, Austria; 3 Clinical Division of Palliative Care, Department of Internal Medicine I, Medical University of Vienna, Währinger Gürtel 18-20, 1090 Vienna, Austria

**Keywords:** Glioblastoma multiforme, Elderly patients, Neurosurgical resection, Hypofractionated radiotherapy, Chemotherapy, Comprehensive geriatric assessment

## Abstract

**Purpose of Review:**

The incidence of glioblastoma multiforme (GBM) increases with age; more than half of newly diagnosed patients are older than 65 years. Due to age-dependent decreasing organ functions, comorbidities, functional decline, and increasing risk of social isolation, not all patients are able to tolerate standard therapy of GBM with 6 weeks of radiochemotherapy.

**Recent Findings:**

A set of alleviated therapies, e.g., chemotherapy or radiotherapy alone, hypofractionated radiotherapies with different total doses and variable fractionation regimens as well as hypofractionated radiotherapy with concomitant and adjuvant chemotherapy, have been evaluated during the last years. However, clinicians are still unsure which therapy would fit best to a given patient. Recently, the predictive value of comprehensive geriatric assessment regarding tolerance of chemotherapy and prediction of early mortality has been validated for older GBM patients in a retrospective trial.

**Summary:**

Thus, it appears that neuro-oncology is now ready for the prospective implementation of geriatric assessment to guide treatment planning for elderly GBM patients.

## Introduction

In the mid-nineties of the last century, in the planning phase of the (European Organisation for Research and Treatment of Cancer) EORTC 26921/NCIC 03 trial of concomitant chemoradiation and adjuvant chemotherapy for patients with newly diagnosed GBM [1,2], the median age of GBM patients in trials used to be 50 to 58 years [3–5]. The upper patients’ age limit of 70 years for the trial was not a matter of controversial debates during the trial planning meetings of the EORTC brain tumor group. Still, this trial defined the standard of care for patients with newly diagnosed GBM. Age-dependent analysis of patient outcomes showed that the benefit of this regimen declines continuously with age, so that the extrapolation of this treatment to elderly patients is questionable [1, 2].

Meanwhile, life expectancy increased while mortality for other reasons declined and the availability of imaging diagnosis increased worldwide. The median age of patients with newly diagnosed GBM is 64 years according to data from the USA, France, and the Austrian Brain Tumor Registry [6–9]. During the last decades, since it became apparent that oncologic treatment regimens developed for and tested in adult patients are not necessarily suitable for elderly patients with variable comorbidities, social and cognitive limitations, and age-dependent declining organ functions, a wide range of tools for assessing the resources and limitations of elderly persons have been developed. These tools allow testing of elderly GBM patients and to allocate them into three groups: fragile elderly patients, aged over 85 years, or patients with severe comorbidities and/or dependencies in activities of daily living (ADL) and instrumental activities of daily living (IADL), vulnerable patients with some comorbidity or an isolated dependence, and fit elderly who show normal functionality and are able to manage their lives independently [10–12].

For elderly patients with GBM, the situation is even more complex, as they are affected simultaneously by a malignant tumor and a neurodegenerative disease, leading to progressive neurological deficits and loss of cognitive functions and thus impacting self-care and decision-making abilities [13, 14]. Planning a complex treatment for patients with high neurological symptom burden and/or cognitive deficits is a common challenge for neuro-oncologists. However, the formal inclusion of geriatric assessment tools or geriatric consultations as part of treatment assignment has not yet entered neuro-oncology. Like in other tumor entities, there has been a lack of trial participation for elderly glioma patients in the past while numbers of relevant studies have increased in recent years [15•, 16•, 17–21]. However, none of these trials included a geriatric assessment, leaving clinicians still unsafe on how to choose an optimal treatment for a given patient. Therefore, we will very briefly review and comment the actual situation for treating elderly patients with GBM.

## Epidemiology and Risk Factors

The US Registry data show that the incidence of GBM increases with age, rising from 0.15 per 100,000 population per year in children to a peak of 15.03 per 100,000 aged 75 to 84 years [7]. This means that nearly half of the patients are diagnosed with GBM aged 65 years or more and are thus designated as elderly patients. However, there is of course no definition of “elderly” with a clear cut calendar age limit as reflected by the different age limits in the studies ranging from 55 to 70 years [15•, 16•, 17–21].

Only very few patients (1–2%) with GBM are affected by a hereditary cancer syndrome like Li-Fraumeni syndrome, Turcot syndrome, and neurofibromatosis type 1 or 2, and usually develop GBM earlier in life. The main number of patients is sporadic GBM. GBM incidence seems to increase with age, potentially a result of cumulative exposure to still unidentified noxes and stressors. The only so far identified cause is ionizing radiation [22], as was most convincingly shown by a population-based study including more than 10,000 persons treated with low-dose irradiation for tinea capitis that showed a dose-dependent increased risk of 1.98/Gy (95% CI 0.73–4.69) [23, 24]. Long-term survivors from childhood brain tumors treated with radiotherapy also show an increased risk for the development of malignant gliomas [25]. Moreover, as sequences of human cytomegalovirus can be found in almost all malignant gliomas, an oncomodulatory role of the cytomegalovirus or of other yet unidentified viral interactions can be postulated. How this could be explored further or exploited for future therapy remains a matter of ongoing debate and research [26].

## Molecular Characteristics of Glioblastoma in the Elderly

Genome-wide genetic and epigenetic sequencing of GBM cases within the Cancer Genome Atlas shows that GBM in the elderly usually lacks the prognostic favorable features of (isocitrate dehydrogenase) IDH mutation and of the glioma CpG island methylator phenotype (G-CIMP). Nevertheless, MGMT promoter methylation was found in near half of GBM diagnosed in the elderly [18••, 27]. In GBM of elderly patients, more neoangiogenesis in response to hypoxia was described [28] and expression of higher levels of vascular endothelial growth factor than in tumors from adults [29].

## Surgery

The impact of maximal safe cytoreduction is demonstrated by the meta-analysis of Almenawer et al. in grouping data from 34 studies published from 1991 to 2014 on a total of 12,607 GBM patients aged 60 years and older. Patients that underwent gross total resection survived in median for 14 months, patients with subtotal resection for in median 8.7 months, and patients who were biopsied for 5.7 months [30•]. There is only one prospective, randomized trial comparing biopsy to maximal feasible resection and this trial was terminated early after 30 patients, as patients who underwent a biopsy survived for in median 2.8 months (55–157 days) and patients after resection for in median 5.6 months (146–278 days) [31•]. These results are clear enough to answer this question definitely. By comparing Karnofsky performance status scales (KPS) before and after neurosurgical intervention, Almenawer’s meta-analysis also provides evidence that, besides a gain in survival time, the functional status of elderly patients improves after resection while it remains stable or deteriorates after biopsy only.

Regarding the amount of postoperative complications after neurosurgery in elderly persons after neurosurgical procedures, there is a report from Chibbaro et al. who analyzed the number of procedures performed, the length of hospital stay, and mortality within 1 month at a university hospital in Paris over a 25-year period [32]. The number of neurosurgical interventions in elderly patients increased steeply from 1983 to 1985 from 10% of patients treated to 24% in the period of 2003 to 2005. The length of hospital stays of the elderly decreased from 23.6 to 11.2 days and the mortality rate after tumor resection decreased from 40 to 33%. All these rates are much higher than the equivalent rates for adult patients. Newer data from a single-center analysis including 178 elderly patients with GBM found comparable survival rates in different stages of age and those being strongly related to preoperative KPS, eloquence of tumor origin, and extent of resection [33].

## Radiotherapy

Radiotherapy of malignant gliomas dates back more than eight decades. The actual dosing scheme with daily fractions of 1.8 to 2 Gy up to a dose of 60 Gy was established by an early “meta-analysis” of three consecutive studies conducted by the Brain Tumor Study Group from 1966 to 1975, pooling the data of 621 patients [34••, 35]. Interestingly, there was no upper age limit (nor exclusion of pediatric patients) in these studies, but the median age was 57 years. The deplorably short median survival times of elderly GBM patients of only 4 months, the frequency of exhaustion and fatigue, difficulties to logistically comply with the 6-week radiotherapy course, and the high frequency of severe cognitive deficits resulted in a controversial discussion of the benefit of radiation in elderly brain tumor patients [36, 37].

The French Association of Neuro-Oncology has conducted a landmark trial that prospectively randomized compared patients aged 70 years or more who presented after GBM resection with a KPS of at least 70%, thus able to live independently receiving best supportive care only versus radiation therapy (50 Gy, 1.8 Gy/ fraction) [15•]. This trial was terminated at the first interim analysis with 85 patients, as radiation therapy nearly doubled the survival time from 3.75 months (16.9 weeks) in the best supportive care group to 6.5 months (29.1 weeks) in the RT group.

A second landmark study was performed by Roa et al. in Canada who compared the standard radiation schedule of 30 fractions with 2.0 up to 60 Gy to a hypofractionated RT schedule of 40 Gy in 15 fractions, limiting the duration of radiotherapy to 3 weeks [16•]. The overall survival for patients treated with the standard schedule was 5.1 months compared with 5.6 months in the hypofractionated arm. The randomized Atomic Agency phase III trial conducted in fragile elderly patients aged more than 50 years or patients aged at least 65 years explored the hypofractionated schedule of 40 Gy/15 fractions and a short course RT consisting in 5 fractions of 5 up to 25 Gy lasting only 1 week [17]. Survival reached 6.4 months for the hypofractionated scheme and 6.8 moths for the extremely short course (de Castro). Due to concerns about radiation toxicity with a high single dose of 5 Gy, this approach was controversially debated and not widely adopted.

The Nordic GBM trial was conceived for patients aged over 60 years that were considered as not fit enough to be treated with standard chemoradiation therapy [18••]. Here also, survival was longer in the patients treated in the hypofractionated arm with 10 fractions at 3.4 up to 34 Gy than in patients receiving standard radiation therapy with 30 × 2 Gy. Of note, as well in the trial by Roa [16•], Malmstöm [18••], and the retrospective analysis by Minnitti of 127 patients, elderly patients treated with 30 × 2 Gy experienced more side effects and needed higher doses and longer treatments with steroids than patients treated with hypofractionated radiotherapy schemes [38]. This led to a guideline by Cabrera et al. recommending hypofractionated radiotherapy for elderly patients with GBM who would not be able to tolerate the standard radiation course of 6 weeks [39].

These trials were exploring “monotherapies” in elderly patients with GBM that were estimated as not fit enough to tolerate the standard 6 weeks of radiochemotherapy. Later analyses of the data that included data on MGMT promoter methylation allowed to conclude if a “monotherapy” with either radiation or chemotherapy for elderly GBM patients is planned, patients with tumors with methylated MGMT promoter would benefit from alkylating chemotherapy with temozolomide whereas patients with tumors with unmethylated MGMT promoter should undergo radiotherapy alone, preferentially a hypofractionated scheme [40, 41]. However, two patterns of care analyses on the US Brain Tumor Registry data show a slightly shorter survival in patients treated with hypofractionated radiotherapy regimen than in elderly patients treated with standard radiation. Both manuscripts discuss that for many years patients who received hypofractionated RT were older and in worse performance status than those receiving standard adult care that obviously were selected for this treatment because of their clinical fitness [42, 43].

## Chemotherapy-Systemic Therapy

There are no other drugs available for systemic treatment of elderly glioma patients than for adults. Thus, the spectrum of drugs that has shown efficacy is limited to alkylating agents such as temozolomide and lomustine—and less often used fotemustine as an intravenously used compound and BCNU wafers for the intraoperative implantation into the resection cavity. Temozolomide is a usually well-tolerated oral chemotherapeutic agent. The most prevalent side effects are fatigue that may severely affect elderly persons and hematologic side effects which also are more frequent and more severe in elderly [18••, 19••, 44] than in adult patients. The prolonged chemotherapy cycle duration of 6-week interval between two consecutive doses of lomustine is a double-edged sword: on one hand, this therapy is very convenient to apply while on the other hand, the rigidity of the dose levels with capsules of 40 mg and late occurrence of hematologic side effects preclude its use in pretreated, vulnerable, or fragile elderly patients.

## Bevacizumab

The observation of prominent neoangiogenesis and high expression levels of VEGF in GBM of elderly patients provides a rationale for the symptomatic use of bevacizumab, a monoclonal anti-VEGF antibody [45, 46]. Several subgroup analyses of studies including adult and elderly patients showed a trend to a prolonged progression-free survival (PFS) and an increased period of steroid independence in elderly persons and perhaps also on overall survival (OS) while a randomized trial of first-line use of bevacizumab combined to hypofractionated radiotherapy failed to show a survival advantage [21].

## Radiochemotherapy

Using radiochemotherapy in elderly GBM patients by choosing a hypofractionated radiotherapy regimen was studied by the Canadian lead CCTG CE6 trial presented at the plenary session of the American Society of Clinical Oncology (ASCO) 2017. This study compared a hypofractionated radiotherapy scheme of 40 Gy/15 fractions alone to the same RT with concomitant 75 mg/m^2^ temozolomide followed by up to 12-cycle adjuvant temozolomide in 562 randomized patients aged 65 years or older [20••]. The patients admitted into this study were 65 years or older with a KPS of at least 70% and intact metabolic functions.

The combined therapy showed prolonged PFS and OS for all patients, as well in patients whose tumors showed MGMT promoter methylation (median OS 13.5 vs 7.7 months, HR 0.53) and in patients with unmethylated MGMT promoter (OS 10.0 vs 7.9 months, HR 0.75). This is the first study showing a benefit of alkylating therapy in patients with unmethylated MGMT promoter; however, the method to determine MGMT promoter methylation chosen for this study, e.g., methylation-specific PCR, was shown to have problems with the reproducibility of results [47].

Nevertheless, the concept of radiochemotherapy with hypofractionated RT was proven as feasible in this study and is widely used. A single-center retrospective study from Japan reported treatment results from 30 patients aged ≥ 75 years treated with hypofractionated RT and either temozolomide or bevacizumab and temozolomide with a median duration of survival of 12.9 months. The median duration until a deterioration of KPS below 60% was 7.9 months. Of note, leukopenia grade III and IV occurred in 50% of participants during the short concomitant phase [44].

An analysis of the patterns of care in patients with newly diagnosed GBM of the National Cancer Data Base (NCDB) covering data from 38,862 patients diagnosed from January 1998 up to December 2012 showed that the concept of radiochemotherapy was adopted rapidly after the publication of the EORTC 26981/NCIC 03 trial [43]. The use of radiochemotherapy in elderly GBM patients increased approximately 50-fold continuously, whereas the proportion of elderly patients treated up to 60 Gy/30 fractions declined.

A network meta-analysis including any post-surgical therapy by analyzing the outcomes of 1569 patients with newly diagnosed GBM treated within 7 RCTs was recently published and identified hypofractionated RT with concurrent and adjuvant temozolomide as the best treatment option [48•].

## Treatment After Progression

To date, there is no recommendation of a standard treatment for recurrent GBM and even less for elderly GBM patients. However, as the numbers of patients having received multimodal first-line treatment and who present with relapsed diseases with preserved functional status, this question gains actuality. The current practice is to re-evaluate all treatment modalities used in first line for the individual benefit when used in the particular recurrence. In small recurrent GBM, radiosurgery presents a valuable alternative to surgical resection [49].

The French neuro-oncological society conducted a retrospective study to assess survival per age and per treatment in recurrence in patients aged at least 70 years at initial GBM diagnosis and had received neurosurgical resection, followed by radiation and concomitant and adjuvant chemotherapy with temozolomide and who relapsed between 2005 and 2015 in comparison with adult patients treated at the same institution within the same time period [50]. A total of 117 elderly patients were enrolled in this study. Compared with adult patients, less patients aged over 70 years received treatment in recurrence than in the adult patient group (85/116, 73% of elderly patients vs 575/661, 87% of adult patients, *p* < 0.001). All treatment modalities and symptomatic anti-angiogenic treatment with bevacizumab were less employed in the elderly.. Of note, those elderly patients who received any therapy in relapse responded with the same rate and for same time periods as adult patients.

## Geriatric Assessment in Patients with Glioma

Standardized, multidimensional evaluation of individual risk factors and also of individual resources of an elderly person about to start cancer treatment, e.g., comprehensive geriatric assessment (CGA), most optimally followed by a geriatric intervention, has been introduced in oncology since the turn of the millennium [10]. There are yet many tumor entities where trials implicating CGA have been conducted and showed benefits by tailoring adequate treatment to elderly patients. Also, in neuro-oncology, there is now at least one trial using a geriatric screening tool in the enrollment phase for elderly patients. Lombardi et al. reported a retrospective analysis of a single-center study administering CGA to patients with newly diagnosed GBM and its validation as a predictor of mortality [51••]. This study included 113 patients aged 65 to 84 years (median 72 years). According to CGA, respectively one-third of the patients were classified as fragile elderly with a median survival of 10.3 months (95% CI 8.8–11.8 months), vulnerable with a median survival of 12.1 months (95% CI 8.1–16.1 months) and fit patients who survived in median for 16.5 months (95% CI 1.6–18.2 months). The treatment allocations were left to the treating neuro-oncologists. Of note, 98% of fit patients were treated with combined radiochemotherapy, as also 90% of the vulnerable and 52% of the fragile elderly patients. Patients with a KPS between 40 and 60% mainly received monotherapies; either temozolomide alone when the MGMT promoter of their GBM was methylated or hypofractionated radiation alone in case of unmethylated MGMT promoter. Of note, fragile elderly patients received in average three adjuvant treatment cycles, whereas vulnerable and fit patients received five adjuvant chemotherapy cycles, respectively.

## Supportive Care

All symptomatic treatments that are indicated in adult patients are of course also adequate for elderly GBM patients. This includes the management of tumor edema and seizures as well. More than in adult patients, attention has to be payed to the abilities of the patients to comply with the treatment, mastering to keep the appointments of radiotherapy and respecting the dose and schedule of oral chemotherapy. Furthermore, medications needed against edema and seizures in addition to potential medications needed against comorbidities have to be considered. Moreover, considering the abovementioned aspects supports patients to maintain an ordinary life during the treatment period and thereafter [52••]. Geriatric assessment and geriatric counseling allow timely detection of problems that lead to potential toxicities, treatment discontinuations, or organization failures, and to precautionary address them. The concept of early introduction of palliative care is appealing for elderly persons with GBM.

## Future Directions

The ASCO mandates to examine patients aged 65 years and older undergoing cancer treatment with a geriatric assessment in order to detect vulnerabilities that are not apparent in normal oncologic routine [53••]. Of note, such an approach applied by Hamaker et al. in the Netherlands identified a geriatric syndrome in > 90% of a cohort of elderly patients [54••]. Half of them were undetected before the assessment. Oncologic therapy was modified in 80% of the assessed patients; of note, therapy was intensified and alleviated in nearly half of the patients, respectively. Therefore, a geriatric assessment helps to individualize the therapy for each person. Nutritional interventions and logistic support may enable some patients to undergo oncologic therapy under safe conditions.

However, the benefit of geriatric assessment–based approach to neuro-oncologic patients has still to prove its efficacy in avoiding toxicity and early therapy-induced deteriorations in health status. This should allow patients to survive in good performance status as long as possible. Interestingly, the topic of treating GBM in the elderly has been intensely reviewed during the last years, reflecting that choosing therapy for elderly GBM patients was indeed perceived as a problem. All therapeutic options remind on the importance of appropriately balancing quality of life with burden and benefits of therapies [55–62, 63•]. While some studies claim for a common definition for elderly [15, 40, 57, 58] or describe and evaluate different treatment patterns retrospectively [42, 59, 60] or by pattern analysis [48•, 57], some base their treatment recommendations on scoring age, KPS, and co-morbidity [55, 57]. Most studies acknowledge the inherent diversity of elderly patients with its diversity between calendar age and biological age [60 – 62, 63•] while a few of them recommend the use of geriatric assessment to guide treatment assignment for elderly patients with GBM [56, 60, 63]. Finally, the last studies of the EORTC Brain Tumor Group include a geriatric screening tool for patients aged 65 years and older, showing that neuro-oncology is ready to incorporate geriatric assessment into the treatment planning for elderly patients [64]. Logical next steps include to perform a geriatric assessment for guiding treatment choice in elderly patients with glioblastoma and to include further CGA regularly during follow-up.
